# Abnormal expressions of AGEs, TGF-β1, BDNF and their receptors in diabetic rat colon–Associations with colonic morphometric and biomechanical remodeling

**DOI:** 10.1038/s41598-018-27787-2

**Published:** 2018-06-21

**Authors:** Hong Sha, Xiaolin Tong, Jingbo Zhao

**Affiliations:** 1 Institute of Clinical Medical Sciences, China-Japan Friendship Hospital, Beijing, 100029 China; 2 Guang’anmen Hospital, China Academy of Chinese Medical Sciences, Beijing, 100053 China; 3 Department of Clinical Medicine, Aarhus University, 8200 Aarhus N, Denmark

## Abstract

Present study aims to investigate the role of AGEs, TGF-β1, BDNF and their receptors on diabetes-induced colon remodeling. Diabetes was induced by a single tail vein injection 40 mg/kg of STZ. The parameters of morphometric and biomechanical properties of colonic segments were obtained from diabetic and normal rats. The expressions of AGE, RAGE, TGF- β1, TGF- β1 receptor, BDNF and TrkB were immunohistochemically detected in different layers of the colon. The expressions of AGE, RAGE, TGF-β1 and TGF- β1 receptor were increased whereas BDNF and TrkB were decreased in the diabetic colon (P < 0.05, P < 0.01). AGE, RAGE and TGF-β1 receptor expressions were positively correlated whereas the BDNF expression was negatively correlated with most of the morphometry and biomechanical parameters (P < 0.05, P < 0.01, P < 0.001). AGE, TGF- β1 and BDNF in different layers correlated with their receptors RAGE, TGF- β1 receptor and TrkB respectively. STZ-induced diabetes up-regulated the expression of AGE, RAGE, TGF- β1 and TGF- β1 receptors and down-regulated BDNF and TrkB in different layers of diabetic colon mainly due to hyperglycemia. Such changes maybe important for diabetes-induced colon remodeling, however it is needed to further perform mechanistic experiments in order to study causality or approaches that explain the relevance of the molecular pathways.

## Introduction

A previous study has demonstrated that experimental diabetes could induce colon morphological and biomechanical remodeling^1^. Following the development of diabetes, the colonic wall became thicker and the stiffness of the wall increased in a time-dependent manner. Such remodeling may play an important role in diabetic GI complications, including constipation^2^. However, the molecular pathways of diabetes-induced colon remodeling are not well understood.

Advanced glycation end products (AGEs) are formed physiologically and s accelerated in diabetes^3^. AGEs can lead to structural and functional changes by direct interaction with target protein or through their receptor (RAGE)^4^. AGEs and RAGE have been demonstrated to play an important role in diabetic complications including the complications of the gastrointestinal (GI) tract^5^. TGF-β1 is considered to be a core factor in the development of diabetic nephropathy (DN)^6^. In recent years, the relationship between TGF-β1 and other diabetic complications has been gradually brought to attention^7,8^. Some studies have demonstrated that an association exists in the expressions of AGE/RAGE and TGF-β1 during the development of diabetes^9,10^. Active TGF-β1 signaling is transmitted through TGF-β1 receptor and the activated TGF-β1 receptor propagates intracellular signaling by recruiting and phosphorylating receptor-regulated Smad proteins^11^. Neurotrophic factors (NTFS) are polypeptides or small proteins that support and enhance the growth, differentiation, and survival of neurons^11^. Brain derived neurotrophic factor (BDNF) is a member of NTFS^12^. Tropomyosin receptor kinase B (TrkB) is a receptor for BDNF^13^. BDNF and TrkB are broadly expressed in the brain and the peripheral nervous system^14–16^. BDNF/TrkB-stimulated intracellular signaling is critical for neuronal survival, morphogenesis, and plasticity^17^. When BDNF binds to the TrkB receptor, it results in the recruitment of proteins that activate three different signaling pathways: Ras/MAPK-ERK pathway, PI3-K pathway and PLC pathway^17^. Reduced levels of BDNF have been evidenced in diabetic patients^18^ and may be associated with diabetic complications^19,20^. It has been shown that BDNF is highly expressed in the colon^21^. However, no data on the relation between BDNF/TrkB and diabetic colon remodeling has been reported so far.

In order to investigate whether AGE, RAGE, TGF- β1 and their receptors play a role in the colon remodeling induced by diabetes, the expressions of AGE, RAGE, TGF- β1, TGF- β1 receptor, BDNF and TrkB were detected in the colon wall of streptozotocin (STZ)-induced rats. Furthermore, the association between the expression of these proteins and the histomorphometric and biomechanical parameters were analyzed.

## Results

### General data, morphometry data and biomechanical data

The general data, morphometry data and biomechanical data obtained from our previous publication^22^ are shown in Table 1. The blood glucose level was about 4-fold higher in the Diabetes group compared with that of the Control group (p < 0.01). The body weight in the Diabetes group was nearly 50% lower than that in the Control Group (p < 0.01). The wet weight per unit length, wet-weight to body weight ratio, no-load wall thickness, and cross-section wall area of the colonic segments were significantly higher in the Diabetes group compared with those of the Control group (p < 0.01). The opening angles were significantly higher in the Diabetes group compared with those in the Control group (p < 0.01). A similar trend was found for the inner and outer residual strains; i.e., the absolute values of the residual strain were significantly higher in the Diabetes group compared with those in the Control group (p < 0.05, p < 0.01). Computation of constant a showed a significant difference between the Diabetes group and the Control group (p < 0.01).

**Table 1:** Glucose, body weight, morphometry and biomechanical parameters of colon.

	Control	Diabetes
Blood glucose (mmol/L)	5.06 ± 0.04	30.23 ± 0.41**
Body weight (g)	443.69 ± 5.01	231.22 ± 5.05**
Wet weight per unit (g/cm)	0.13 ± 0.02	0.15 ± 0.02*
Wet weight per unit to Body weight ratio	0.00027 ± 0.00003	0.00071 ± 0.00017**
Wall thickness (mm)	0.98 ± 0.05	1.23 ± 0.04**
Wall area (mm^2^)	11.04 ± 0.81	16.55 ± 0.98**
Opening angle (degree)	91.29 ± 8.02	237.12 ± 26.34 **
Inner residual strain (unitless)	-0.21 ± 0.02	-0.29 ± 0.03 *
Outer residual strain (unitless)	0.12 ± 0.02	0.22 ± 0.03 **
Circumferential constant a (kPa)	1.03 ± 0.16	2.28 ± 0.29 **
Longitudinal constant a (kPa)	19.38 ± 3.82	50.15 ± 8.55 **

Compared with Con: *P < 0.05, **P < 0.01.

### Fractions of AGE, RAGE, TGF-β1, TGF- β1 receptor, BDNF and TrkB between two groups

**Figure 1:**
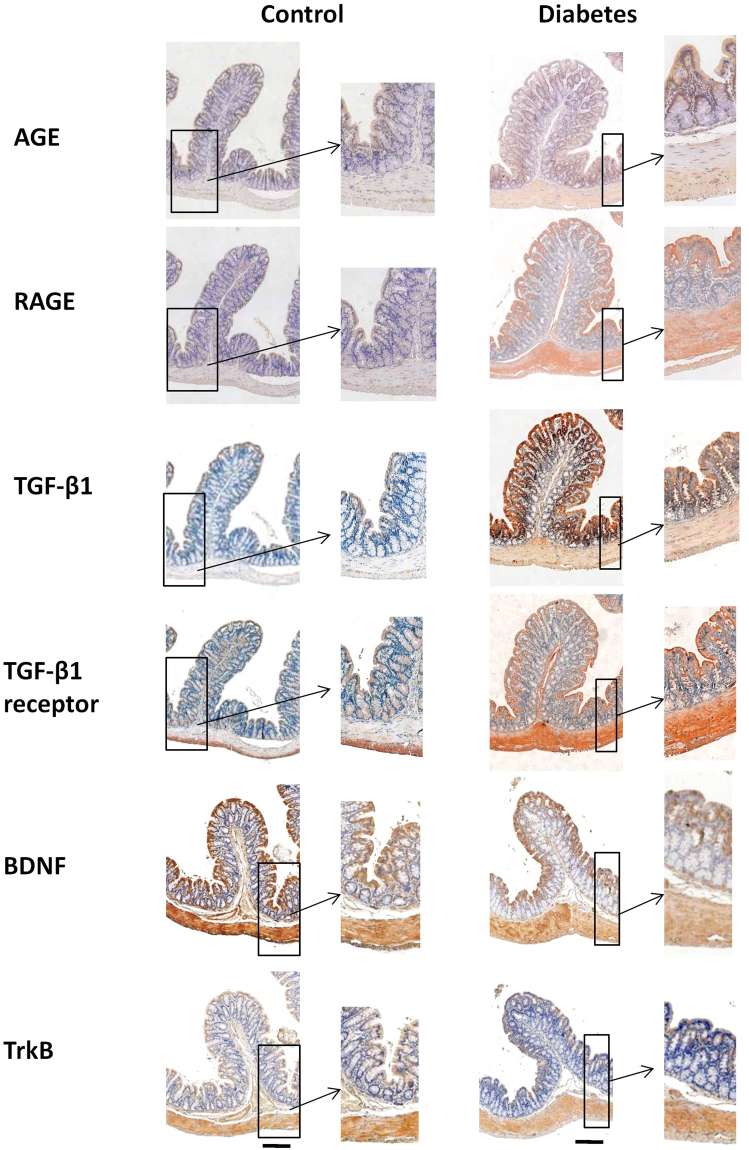
The representative samples of immunohistochemical staining for AGE, RAGE, TGF-β1, TGF- β1 receptor, BDNF and TrkB in the colon wall of two groups. The microscopy with high magnification have been inserted in each single histological photo (arrow) in order to display the localization of markers. The staining of all proteins was stronger in the muscle layer than other layers. In the different layers, the staining of AGE, RAGE, TGF-β1 and TGF- β1 receptor was stronger whereas the staining of BDNF and TrkB was weaker in the Diabetes group than in Control group. Bar = 100 um.

**Figure 2:**
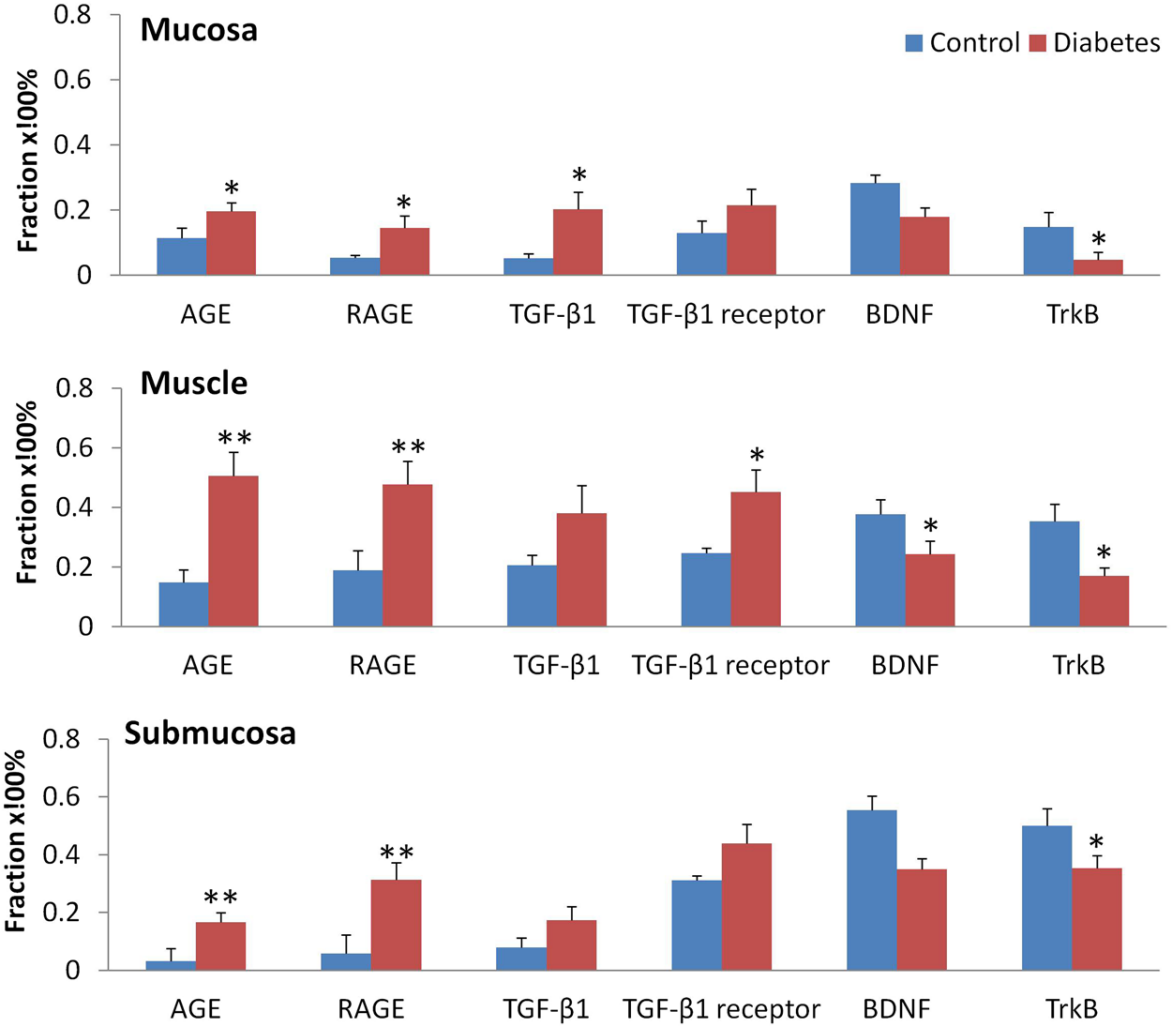
The fraction of AGE, RAGE, TGF-β1, TGF- β1 receptor, BDNF and TrkB in the different layers of the colon between two groups. In the different layers, the fraction of AGE, RAGE, TGF-β1 and TGF- β1 receptor was bigger whereas the fraction of BDNF and TrkB was smaller in the Diabetes group than in Control group. Compared with Control group: *P < 0.05, **P < 0.01.

The representative samples of immunohistochemical staining is shown in Fig. 1 and the fraction of AGE, RAGE, TGF-β1, TGF- β1 receptor, BDNF and TrkB in the different layers of the colon between two groups are shown in Fig. 2. Generally, the expression of all proteins was stronger in the muscle layer than other layers. In different layers of colon wall, the expressions of AGE, RAGE, TGF-β1 and TGF- β receptor were stronger whereas BDNF and TrkB expressions were weaker in the Diabetes group than in Control group. Significant difference were found for AGE, RAGE and TrkB for all three layers (P < 0.05, P < 0.01), TGF- β1 in mucosa layer (P < 0.05), and TGF- β1 receptor and BDNF in muscle layer (P < 0.05).

### Correlation analysis results

#### Single *linear* correlation analysis

It was shown that the glucose level was associated with the most parameters of morphometry, biomechanical properties and the expressions of all proteins studied in different layers. In relation to morphometry parameters, the most significant correlation was found for the body weight and the wet weight per unit length of the segments to body weight ratio (P < 0,001). In relation to the biomechanical parameters, besides inner residual strain, all other parameters were significantly correlated with the glucose levels (P < 0.05, P < 0.01). In relation to the expressions of all proteins studied, glucose levels were strongly correlated with the AGE and RAGE expressions in different layers (P < 0.05, P < 0.01). The glucose levels were also significantly correlated with TGF-β1 in mucosa layer, TGF- β1 receptor in muscle layer, BDNF in muscle layer and TrkB in mucosa and submucosa layers (P < 0.05). The details of correlation equations and values of R and P are shown in Table 2.

**Table 2:** The relation between glucose levels with other parameters.

Parameters		Linear regression equation	R value	F value	P value
Morpho-metry	BW	BW = 467.941 − (7.606 * Glu)	0.92	49.364	<0.001
	Wt	Wt = 0.110 + (0.00148 * Glu)	0.717	9.527	0.013
	Wt/BW	Wt/BW = 0.000193 + (0.0000164 * Glu)	0.86	25.562	<0.001
	WH	WH = 0.936 + (0.00904 * Glu)	0.666	7.162	0.025
	WA	WA = 9.402 + (0.241 * Glu)	0.775	13.56	0.005
Biome-chanics	Res-in	Res-in = -0.216 − (0.00208 * Glu)	0.461	2.435	0.153
	Res-out	Res-out = 0.0710 + (0.00565 * Glu)	0.648	6.501	0.031
	OA	OA = 66.477 + (5.476 * Glu)	0.809	17.044	0.003
	C-a	C-a = 0.649 + (0.0536 * Glu)	0.736	10.631	0.01
	L-a	L-a = 10.950 + (1.300 * Glu)	0.707	8.983	0.015
AGE	Muc	Muc = 0.101 + (0.00350 * Glu)	0.677	7.62	0.022
	Mus	Mus = 0.0837 + (0.0135 * Glu)	0.767	12.893	0.006
	Sub	Sub = 0.0155 + (0.00465 * Glu)	0.686	8.015	0.02
RAGE	Muc	Muc = 0.0382 + (0.00342 * Glu)	0.582	4.609	0.06
	Mus	Mus = 0.127 + (0.0118 * Glu)	0.754	11.854	0.007
	Sub	Sub = 0.00415 + (0.00999 * Glu)	0.777	13.673	0.005
TGF-β	Muc	Muc = 0.0247 + (0.00593 * Glu)	0.698	9.488	0.012
	Mus	Mus = 0.171 + (0.00692 * Glu)	0.511	3.54	0.089
	Sub	Sub = 0.0854 + (0.00272 * Glu)	0.395	1.845	0.204
TGF-β-R	Muc	Muc = 0.0941 + (0.00441 * Glu)	0.469	2.817	0.124
	Mus	Mus = 0.141 + (0.00908 * Glu)	0.708	10.025	0.01
	Sub	Sub = 0.202 + (0.00567 * Glu)	0.555	4.458	0.061
BDNF	Muc	Muc = 0.290 − (0.00237 * Glu)	0.362	1.507	0.248
	Mus	Mus = 0.577 − (0.00744 * Glu)	0.705	9.869	0.01
	Sub	Sub = 0.406 − (0.00511 * Glu)	0.558	4.518	0.059
TrkB	Muc	Muc = 0.177 − (0.00403 * Glu)	0.593	5.433	0.042
	Mus	Mus = 0.490 − (0.00447 * Glu)	0.498	3.291	0.1
	Sub	Sub = 0.425 − (0.00791 * Glu)	0.68	8.591	0.015

Notes: BW, body weight; Wt, wet weight per unit length; Wt/BW, wet weight to body weight ratio; WH, wall thickness; WA, Wall area; Res-in, inner residual strain; Res-out, Outer residual strain; OA, opening angle, C-a, circumferential constant a; L-a, longitudinal constant a; Muc, mucosa; Mus, muscle; Sub, submucosa; Glu, glucose.

**Figure 3:**
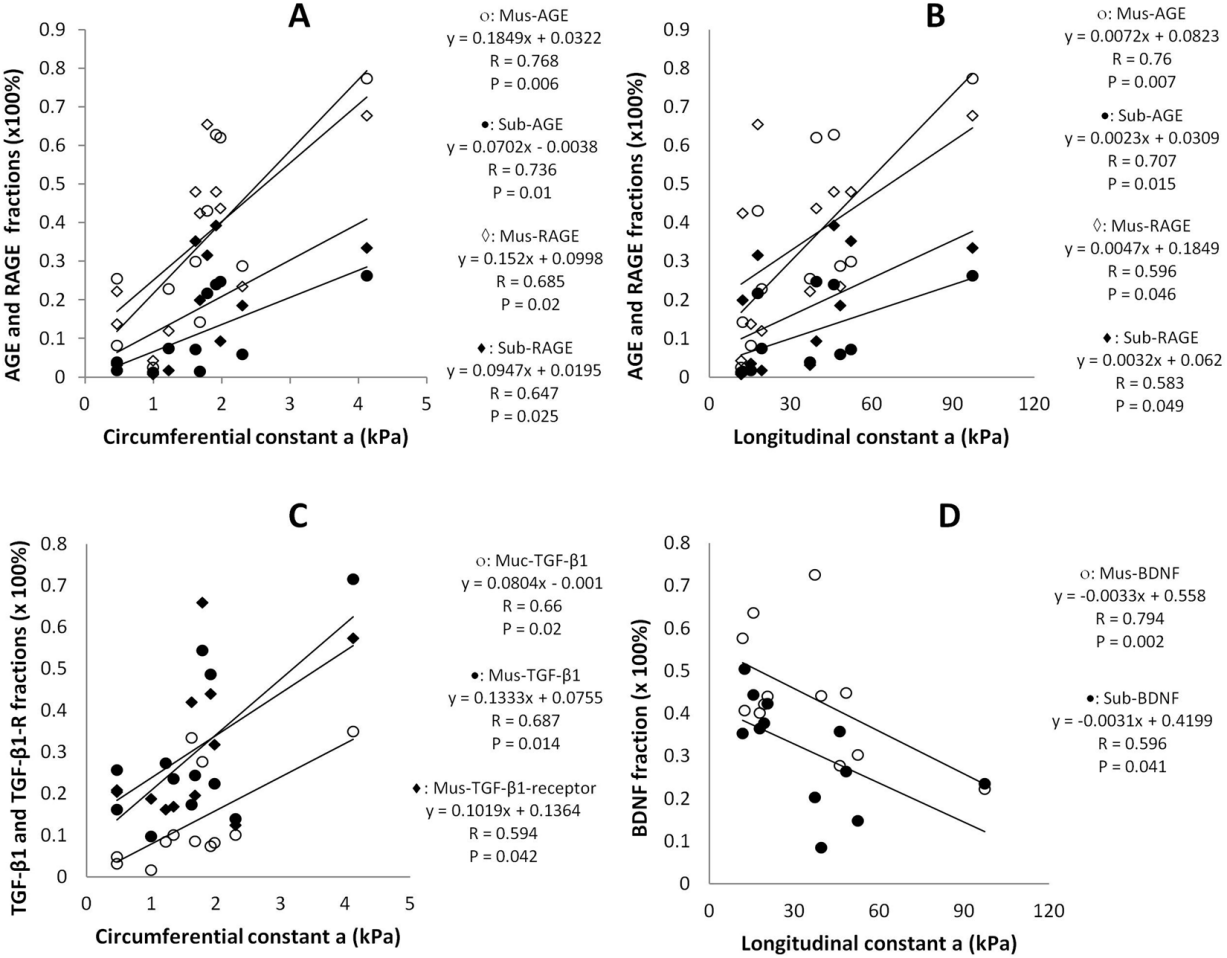
(**A**) Correlation between AGE and RAGE in muscle layer and submucosa layer with circumferential constant a; (**B**) Correlation between AGE and RAGE in muscle layer and submucosa layer with longitudinal constant a; (**C**) Correlation between TGF-β1 and TGF-β1 receptor in mucosa layer and TGF-β1 muscle layer with circumferential and longitudinal material constant a; (**D**) Correlation between BDNF in muscle and submucosa layers with longitudinal constant a.

Table 3 shows the correlation between expressions of AGE, RAGE, TGF- β1, TGF- β1 receptor, BDNF and TrkB with morphometry and biomechanicl parameters. Only significant correlation results (P < 0.05 or close to 0.05) are shown. AGE and RAGE in different layers were significantly correlated with most morphometry and biomechanical parameters. It is especially interesting that AGE and RAGE in the muscle and submucosa layers were positively correlated with circumferential and longitudinal material constant a (Fig. 3 A&B, P < 0.05, P < 0.01). The expression of TGF-β1 in mucosa and muscle layers was also positively and significantly correlated with circumferential and longitudinal material constant a (Fig. 3 C, P < 0.05). TGF-β1 receptor in different layers correlated with most morphometry parameters and Opening angle. Whereas the BDNF in muscle layer negatively correlated with most of the morphometry and biomechanical parameters including circumferential constant a (Fig. 3D, P < 0.05) and in submucosa layer with longitudinal constant a (Fig. 3D, P < 0.05). TrkB in different layers was found to correlate with wet weight per unit length of the segments, wall thickness, opening angle as well as the residual strain. The details of correlation equations and values of R and P are shown in Table 3.

**Table 3:** The relation between expressions of AGE, RAGE, TGF- β1, TGF- β1 receptor, BDNF and TrkB with morphometric and biomechanical parameters.

Proteins		Linear regression equation	R value	F value	P value
AGE	Muc	Muc = 0.310 − (0.000440 * BW)	0.705	8.875	0.015
		Muc = 0.0822 + (169.012 * Wt/BW)	0.622	5.68	0.041
		Muc = −0.0868 + (0.230 * WH)	0.604	5.158	0.049
		Muc = 0.00227 − (0.649 * Res-in)	0.565	4.216	0.07
		Muc = 0.0944 + (0.000429 * OA)	0.561	4.144	0.072
	Mus	Mus = 0.810 − (0.00145 * BW)	0.685	7.963	0.02
		Mus = −0.360 + (5.096 * Wet-W)	0.6	5.059	0.051
		Mus = 0.0453 + (585.792 * Wet-W/BW)	0.635	6.095	0.036
		Mus = −0.484 + (0.745 * WH)	0.577	4.495	0.063
		Mus = −0.291 + (0.0452 * WA)	0.8	15.991	0.003
		Mus = 0.0868 + (1.426 * Res-out)	0.709	9.119	0.014
		Mus = −0.0638 + (0.00237 * OA)	0.913	45.05	<0.001
		Mus = 0.0322 + (0.185 * C-a)	0.768	12.932	0.006
		Mus = 0.0823 + (0.00724 * L-a)	0.76	12.323	0.007
	Sub	Sub = 0.300 − (0.000605 * BW)	0.738	10.787	0.009
		Sub = −0.190 + (2.140 * Wt)	0.652	6.644	0.03
		Sub = −0.0318 + (269.423 * Wt/BW)	0.756	11.997	0.007
		Sub = −0.326 + (0.388 * WH)	0.778	13.792	0.005
		Sub = −0.141 + (0.0176 * WA)	0.805	16.544	0.003
		Sub = −0.00796 + (0.629 * Res-out)	0.81	17.168	0.003
		Sub = −0.0239 + (0.000751 * OA)	0.75	11.557	0.008
		Sub = 2.296 + (10.104 * C-a)	0.736	10.631	0.01
		Sub = 5.462 + (0.384 * L-a)	0.707	8.983	0.015
RAGE	Muc	Muc = 0.254 − (0.000467 * BW)	0.658	6.864	0.028
		Muc = −0.105 + (1.513 * Wt)	0.531	3.541	0.093
		Muc = −0.00805 + (220.533 * Wt/BW)	0.714	9.352	0.014
		Muc = −0.152 + (0.231 * WH)	0.533	3.572	0.091
	Mus	Muc = 0.254 − (0.000467 * BW)	0.658	6.864	0.028
		Sub = −0.358 + (4.020 * Wet-W)	0.645	6.414	0.032
		Sub = −0.0981 + (579.748 * Wet-W/BW)	0.857	24.932	<0.001
		Sub = −0.423 + (0.558 * WH)	0.59	4.794	0.056
		Sub = −0.169 + (0.0261 * WA)	0.63	5.911	0.038
		Sub = 0.00952 + (0.00109 * OA)	0.573	4.402	0.065
		Sub = 0.0343 + (0.0967 * C-a)	0.547	3.849	0.081
	Sub	Mus = 0.803 − (0.00139 * BW)	0.734	10.522	0.01
		Mus = −0.207 + (4.076 * Wt)	0.536	3.637	0.089
		Mus = 0.0675 + (566.304 * Wt/BW)	0.687	8.037	0.02
		Mus = −0.0757 + (0.0307 * WA)	0.608	5.281	0.047
		Mus = 0.0824 + (0.00159 * OA)	0.685	7.963	0.02
		Mus = 0.0998 + (0.152 * C-a)	0.706	8.935	0.015
		Mus = 0.185 + (0.00474 * L-a)	0.556	4.032	0.076
**TGF-β1**	Muc	Muc = 0.340 − (0.000623 * BW)	0.625	6.406	0.03
		Muc = −0.00104 + (0.0804 * C-a)	0.66	7.718	0.02
		Muc = 0.0278 + (0.00300 * L-a)	0.629	6.556	0.028
	Mus	Mus = −0.0827 + (0.0268 * WA)	0.588	5.275	0.045
		Mus = 0.0707 + (0.00137 * OA)	0.686	8.882	0.014
		Mus = 0.0755 + (0.133 * C-a)	0.687	8.956	0.014
		Mus = 0.145 + (0.00434 * L-a)	0.573	4.876	0.052
	Sub	Sub = 0.275 − (0.000420 * BW)	0.519	3.682	0.084
		Sub = 0.0148 + (242.305 * Wt/BW)	0.649	7.287	0.022
		Sub = −0.187 + (0.287 * WH)	0.548	4.284	0.065
**TGF-β1-R**	Muc	Muc = 0.379 − (0.000613 * BW)	0.556	4.465	0.061
		Muc = −0.194 + (2.630 * Wet-W)	0.565	4.68	0.056
		Muc = −0.00310 + (357.749 * Wet-W/BW)	0.704	9.807	0.011
		Muc = −0.284 + (0.409 * WH)	0.572	4.853	0.052
		Muc = −0.111 + (0.0202 * WA)	0.637	6.817	0.026
		Muc = 0.0671 + (0.611 * Res-out)	0.547	4.262	0.066
		Muc = 0.0466 + (0.000775 * OA)	0.557	4.508	0.06
	Mus	Mus = 0.664 − (0.00107 * BW)	0.713	10.343	0.009
		Mus = 0.0640 + (487.180 * Wt/BW)	0.702	9.707	0.011
		Mus = −0.0126 + (0.0225 * WA)	0.52	3.698	0.083
		Mus = 0.0998 + (0.00125 * OA)	0.659	7.665	0.02
		Mus = 0.136 + (0.102 * C-a)	0.554	4.425	0.062
	Sub	Sub = 0.560 − (0.000764 * BW)	0.637	6.836	0.026
		Sub = 0.113 + (387.673 * Wet-W/BW)	0.701	9.684	0.011
BDNF	Mus	Mus = 0.184 + (0.000773 * BW)	0.624	6.371	0.03
		Mus = 0.879 − (3.124 * Wt)	0.597	5.539	0.04
		Mus = 0.626 − (371.476 * Wt/BW)	0.65	7.334	0.022
		Mus = 1.036 − (0.530 * WH)	0.66	7.734	0.019
		Mus = 0.827 − (0.0272 * WA)	0.765	14.114	0.004
		Mus = 0.606 − (0.00100 * OA)	0.641	6.99	0.025
		Mus = 0.641 − (0.120 * C-a)	0.794	17.08	0.002
		Mus = 0.558 − (0.00334 * L-a)	0.564	4.669	0.056
	Sub	Sub = 0.612 + (1.212 * Res-in)	0.648	7.219	0.023
		Sub = 0.420 − (0.00306 * L-a)	0.596	5.506	0.041
TrkB	Muc	Muc = −0.0381 + (0.000426 * BW)	0.534	3.981	0.074
		Muc = 0.205 − (203.828 * Wt/BW)	0.554	4.434	0.061
		Muc = 0.223 − (0.000727 * OA)	0.722	10.914	0.008
	Mus	Mus = 0.720 − (2.218 * Wt)	0.498	3.303	0.099
		Mus = 0.529 − (241.906 * Wt/BW)	0.498	3.297	0.099
		Mus = 0.916 − (0.452 * WH)	0.662	7.79	0.019
		Mus = 0.667 − (0.0182 * WA)	0.601	5.657	0.039
	Sub	Sub = −0.00703 + (0.000863 * BW)	0.632	6.645	0.028
		Sub = 0.456 − (352.306 * Wet-W/BW)	0.559	4.555	0.059
		Sub = 0.665 + (1.558 * Res-in)	0.655	7.526	0.021
		Sub = 0.414 − (0.760 * Res-out)	0.549	4.322	0.064
		Sub = 0.482 − (0.00122 * OA)	0.709	10.133	0.01

Notes: Muc, mucosa; Mus, muscle; Sub, submucosa; BW, body weight; Wt, wet weight per unit length; Wt/BW, Wt to BW ratio; WH, wall thickness; WA, Wall area; Res-in, inner residual strain; Res-out, Outer residual strain; OA, opening angle, C-a, circumferential constant a; L-a, longitudinal constant a.

#### Multiple linear correlation analysis

**Figure 4:**
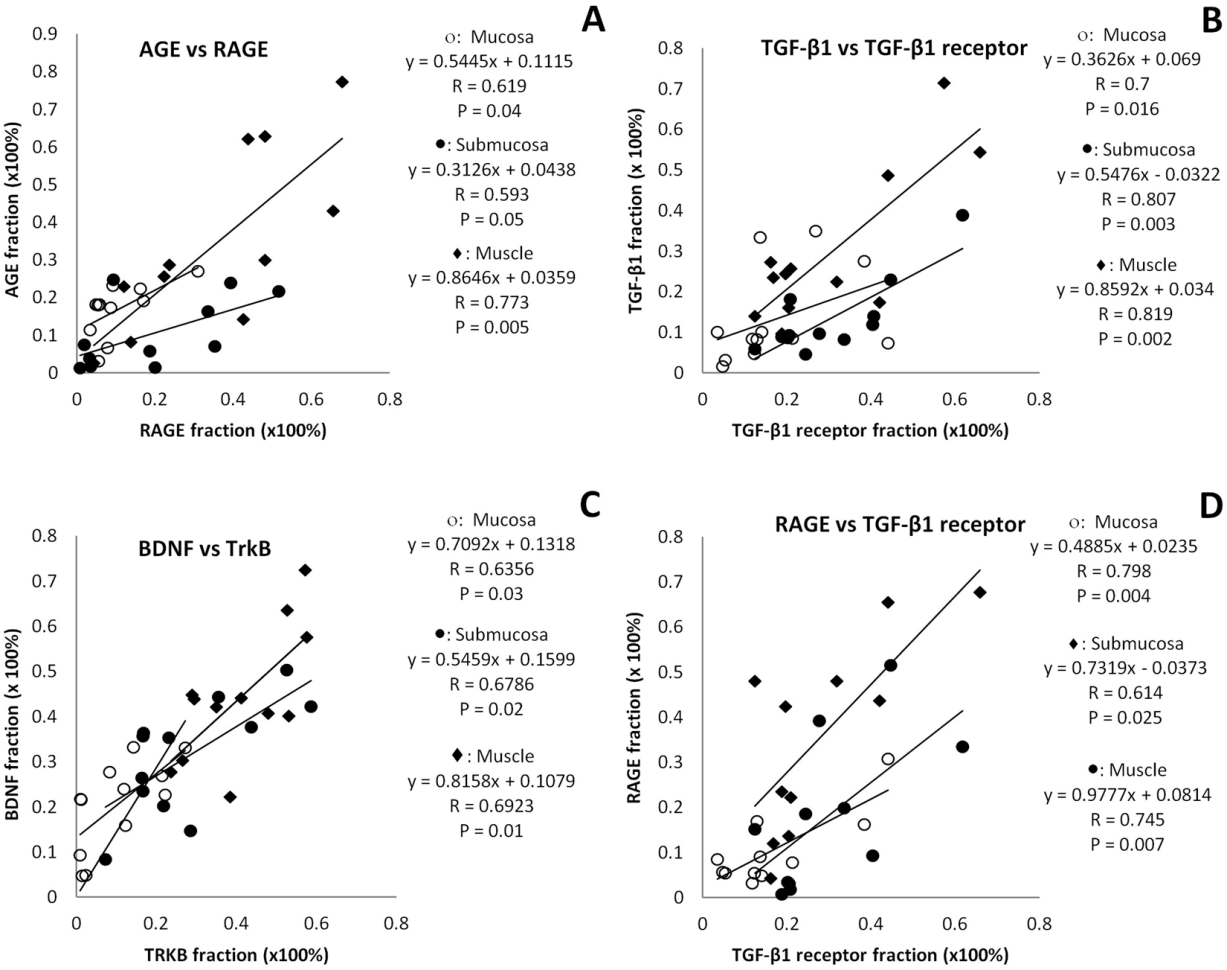
(**A**) Correlation between AGE and RAGE in different layers; (**B**) Correlation between TGF-β1 and TGF-β1receptor in different layers; (**C**) Correlation between BDNF and TrkB in different layers; (**D**) Correlation between RAGE and TGF-β1receptor in different layers.

Interrelation among AGE, RAGE, TGF- β1, TGF- β1 receptor, BDNF and TrkB expressions in different layers are shown in Table 4. AGE, TGF- β1 and BDNF in different layers mostly correlated with their receptors RAGE, TGF- β1 receptor and TrkB (Fig. 4A-C). Similarly, RAGE, TGF- β1 receptor and TrkB in different layers also mostly correlated with AGE, TGF- β1 and BDNF. However, it is interesting to notice that RAGE and TGF- β1 receptor in different layers were strongly correlated with each other (Fig. 4D). The details of correlation equations and values of R, F and P are shown in Table 4.

**Table 4:** Interrelation among AGE, RAGE, TGF- β1, TGF- β1 receptor, BDNF and TrkB expressions in different layers of colon wall.

layers	Multiple linear regression equation	R values	F values	P values	Independent P
Mucosa	AGE = 0.142 + (0.456 * RAGE) + (0.123 * TGF-β1) + (0.0667 * TGF-β1-R) − (0.316 * BDNF) + (0.190 * TRKB)	0.729	1.132	0.447	RAGE 0.073 TGF-β1 0.705 TGF-β1-R 0.864 BDNF 0.567 TRKB 0.722
	RAGE = −0.104 + (0.164 * AGE) + (0.172 * TGF-β1) + (0.337 * TGF-β1-R) + (0.419 * BDNF) − (0.0816 * TRKB)	0.933	6.685	0.029	AGE 0.073 TGF-β1 0.358 TGF-β1-R 0.044 BDNF 0.167 TRKB 0.802
	TGF-β1 = 0.143 + (0.253 * AGE) + (0.988 * RAGE) − (0.000207 * TGF-β1-R) − (0.541 * BDNF) − (0.0205 * TRKB)	0.811	1.928	0.244	AGE 0.705 RAGE 0.358 TGF-β1-R 0.069 BDNF 0.491 TRKB 0.979
	TGF-β1-R = 0.118 + (0.0965 * AGE) + (1.362 * RAGE) − (0.000145 * TGF-β1) − (0.172 * BDNF) − (0.362 * TRKB)	0.904	4.448	0.064	AGE 0.864 RAGE 0.044 TGF-β1 0.069 BDNF 0.798 TRKB 0.569
	BDNF = 0.147 − (0.221 * AGE) + (0.817 * RAGE) − (0.184 * TGF-β1) − (0.0833 * TGF-β1-R) + (0.705 * TRKB)	0.902	4.374	0.066	AGE 0.567 RAGE 0.167 TGF-β1 0.491 TGF-β1-R 0.798 TRKB 0.06
	TRKB = −0.0254 + (0.145 * AGE) − (0.173 * RAGE) − (0.00758 * TGF-β1) − (0.191 * TGF-β1-R) + (0.768 * BDNF)	0.876	3.306	0.108	AGE 0.722 RAGE 0.802 TGF-β1 0.979 TGF-β1-R 0.569 BDNF 0.06
Submucosa	AGE = 0.117 − (0.0408 * RAGE) + (0.388 * TGF-β1) + (0.160 * TGF-β1-R) − (0.116 * BDNF) - (0.273 * TRKB)	0.835	2.309	0.19	RAGE 0.089 TGF-β1 0.529 TGF-β1-R 0.73 BDNF 0.709 TRKB 0.389
	RAGE = −0.0753 − (0.0986 * AGE) + (0.297 * TGF-β1) + (0.932 * TGF-β1-R) − (0.0793 * BDNF) − (0.0953 * TRKB)	0.893	3.933	0.08	AGE 0.089 TGF-β1 0.76 TGF-β1-R 0.051 BDNF 0.87 TRKB 0.852
	TGF-β1 = −0.0617 + (0.216 * AGE) + (0.0686 * RAGE) + (0.395 * TGF-β1-R) + (0.248 * BDNF) − (0.170 * TRKB)	0.921	5.608	0.041	AGE 0.529 RAGE 0.76 TGF-β1-R 0.068 BDNF 0.254 TRKB 0.477
	TGF-β1-R = 0.101 + (0.162 * AGE) + (0.392 * RAGE) + (0.720 * TGF-β1) − (0.110 * BDNF) + (0.228 * TRKB)	0.933	6.77	0.028	AGE 0.73 RAGE 0.051 TGF-β1 0.068 BDNF 0.725 TRKB 0.482
	BDNF = 0.121 − (0.262 * AGE) − (0.0743 * RAGE) + (1.007 * TGF-β1) − (0.245 * TGF-β1-R) + (0.670 * TRKB)	0.795	1.719	0.283	AGE 0.709 RAGE 0.87 TGF-β1 0.254 TGF-β1-R 0.725 TRKB 0.026
	TRKB = 0.0839 − (0.554 * AGE) − (0.0799 * RAGE) − (0.620 * TGF-β1) + (0.455 * TGF-β1-R) + (0.600 * BDNF)	0.841	1.414	0.178	AGE 0.389 RAGE 0.852 TGF-β1 0.477 TGF-β1-R 0.482 BDNF 0.026
Muscle	AGE = 0.0966 + (0.557 * RAGE) + (0.737 * TGF-β1) − (0.0609 * TGF-β1-R) + (0.660 * BDNF) − (1.062 * TRKB)	0.888	3.717	0.088	RAGE 0.037 TGF-β1 0.374 TGF-β1-R 0.931 BDNF 0.452 TRKB 0.204
	RAGE = 0.204 + (0.287 * AGE) − (0.140 * TGF-β1) + (0.631 * TGF-β1-R) − (0.614 * BDNF) + (0.397 * TRKB)	0.929	6.321	0.032	AGE 0.037 TGF-β1 0.745 TGF-β1-R 0.029 BDNF 0.319 TRKB 0.536
	TGF-β1 = 0.0415 + (0.449 * AGE) − (0.165 * RAGE) + (0.409 * TGF-β1-R) − (0.565 * BDNF) + (0.678 * TRKB)	0.895	4.029	0.076	AGE 0.374 RAGE 0.745 TGF-β1-R 0.044 BDNF 0.406 TRKB 0.315
	TGF-β1-R = -0.000233 − (0.0268 * AGE) + (0.539 * RAGE) + (0.296 * TGF-β1) − (0.0810 * BDNF) + (0.194 * TRKB)	0.912	4.942	0.052	AGE 0.931 RAGE 0.029 TGF-β1 0.044 BDNF 0.893 TRKB 0.747
	BDNF = 0.239 + (0.178 * AGE) − (0.321 * RAGE) − (0.250 * TGF-β1) − (0.0496 * TGF-β1-R) + (0.826 * TRKB)	0.926	5.984	0.036	AGE 0.452 RAGE 0.319 TGF-β1 0.406 TGF-β1-R 0.893 TRKB 0.024
	TRKB = −0.0409 − (0.281 * AGE) + (0.204 * RAGE) + (0.295 * TGF-β1) + (0.117 * TGF-β1-R) + (0.812 * BDNF)	0.887	3.705	0.088	AGE 0.204 RAGE 0.536 TGF-β1 0.315 TGF-β1-R 0.747 BDNF 0.024

## Discussion

Diabetic GI complications are common in longstanding diabetes^23^. Poor control of diabetes can affect any segment of the gut including the colon^24^. Although many studies have demonstrated that multiple factors are involved in diabetic GI complications^25^, the mechanisms are not well understood. The main findings found in the present study showed that the expression of AGE, RAGE, TGF- β1 and TGF- β1 receptor was significantly higher whereas the expression of BDNF and TrkB was significantly lower in different colon layers in the Diabetes group than in Control group. The glucose level was associated with the most parameters of morphometry, biomechanical properties and the expressions of all proteins studied in different layers. Furthermore, the expressions of these proteins were highly correlated with the most of the histomorphometric and biomechanical remodeling parameters.

AGEs and RAGE accumulated during the development of DM are associated with cardiovascular complications^26^, retinopathy^27^, nephropathy^28^ as well as GI complications^29^. Chen and co-workers have found that AGE and RAGE are up-regulated in the diabetic rat colon^30,31^. Furthermore, it has been demonstrated that histomorphological and biomechanical remodeling occurred in the colon of diabetic rat model^1,22^. However, the mechanism of such remodeling is not clear. A study on the relation between AGEs and vascular wall stiffness has shown that glycation-induced inter-molecular cross-links contribute to diabetic vascular stiffening^32^. More recently, one study demonstrated that AGEs induced arterial stiffness and aging in a RAGE-dependent manner in mice^33^. Previously we demonstrated that the most histomorphometric and biomechanical parameters of intestinal wall in the GK diabetic rats are associated with the expression of AGE and RAGE^5^. In the present study, we showed that the glucose levels were strongly correlated with the AGE and RAGE expressions in different layers. Diabetes-induced morphological and biomechanical remodeling of rat colon were also found to be associated with the glucose level and abnormal expressions of AGE and RAGE in the different layers of colon wall. Hyperglycemia promotes and accelerates AGE formation. The accumulated AGEs affect the tissue structural changes and neuromuscular functions of diabetic GI tract through receptor-dependent and -independent pathways^34,35^. The former modulates cellular functions through ligation of specific cell surface receptors such as RAGE. The latter alters the extracellular matrix architecture by nonenzymatic glycation and the formation of protein cross-links. Therefore, the abnormal expressions of AGEs and RAGE contribute to diabetes-induced colon remodeling which plays an important role in the GI disorders in diabetes.

Transforming growth factor (TGF)-β1 is a ubiquitously expressed cytokine belonging to a large superfamily of activins/bone morphogenetic proteins^36^. This mediator plays an active role in the processes of wound healing^37^ and the synthesis of ECM molecules^38^. It has been reported that plasma levels of TGF- β 1 are elevated in NIDDM patients and might contribute to the occurrence of diabetic complications^39^. Indeed, many studies have demonstrated that TGF-β1 strongly contributes to diabetic nephropathy^6^, diabetic retinopathy^40,41^ and diabetic neuropathy^42^. In the present study, we demonstrate that the TGF- β1 and TGF- β1 receptor are up-regulated in the diabetic colon wall. Although no detailed molecular pathway has been demonstrated in the present study, the increasing level of TGF- β1 may activate TGF- β1 receptors through ligand binding and subsequently activate Smad proteins through phosphorylation^43^. It has been reported that a significant upregulation of TGF-β1, TGF-β receptors and the effectors p-Smad2/3 in the colon mucosa of diabetic rats^11^. Such deregulation of the TGFβ1 pathway associated with the appearance of myofibroblasts and the accumulation of ECM in the mucosa of diabetic colon. Insulin treatment could attenuate the stimulating effect of diabetes on colon ECM deposition and TGFβ/Smad signaling^11^. Therefore, their data provide the evidence that TGF-β1/Smad is a key component of intestinal tissue remodeling in diabetes. Furthermore, having studies demonstrated the effects of bone morphogenetic protein (BMP2) on promoting enteric neuronal differentiation in culture through a Smad dependent pathway^44^. Using an *in vivo* STZ-induced diabetic model, it has been demonstrated a decrease in the number of myenteric neurons after the onset of diabetes. At same time, BMP/Smad signaling in the myenteric plexus of the diabetic small intestine has also been changed^45^. Therefore, TGF-β1/Smad may be a key component of intestinal tissue remodeling in diabetes. Data lacks in relation to the association between TGF-β1 and TGF-β1 receptor with gastrointestinal morphological and biomechanical remodeling in diabetes. Fleenor and co-workers reported that arterial stiffening with aging is associated with TGF-β1-related changes in adventitial collagen^46^. Few studies demonstrated that TGF- β1 increased F-actin levels in single chondrocytes leading to the stiffening of cells^47^. In the present study we demonstrated that TGF-β1 in muscle layer correlated with circumferential and longitudinal material constant a, whereas TGF-β1 receptor in different layers correlated with most morphological and biomechanical parameters. It is also interesting to note that RAGE and TGF- β1 receptor in muscle layers were strongly correlated each other. It may indicate that either TGF-β1 is an independent contributing factor or TGF-β1 and AGE are co-contributors to the morphological and biomechanical remodeling of colon in diabetes. The detailed molecular pathways for the effects of AGE and TGF-β1 on colonic remodeling in the diabetes need to be explored further.

Brain-derived neurotrophic factor (BDNF) and its receptor TrkB regulate dendritic and axonal growth during development and maintenance of the mature nervous system through different cellular and molecular mechanisms^48^. Decreased levels of BDNF are associated with neurodegenerative diseases with neuronal loss, such as Parkinson’s disease, Alzheimer’s disease, multiple sclerosis and Huntington’s disease^17^. In relation to diabetes, data from animal experiments and human studies suggested that BDNF may contribute to glucose metabolism and plays a pathogenic role in the development of type 2 diabetes mellitus in human^49^. Some studies have found that BDNF levels are lower in individuals with type 2 diabetes compared to non-diabetic individuals both in plasma and serum^18–20,50,51^. The abnormal level of BDNF may be associated with diabetic complications^18–20^. One study demonstrated that in the diabetic rat brains, both protein and mRNA levels of BDNF are severely reduced. These results suggest that diabetic neuropathies in the brain are, at least in part, caused by a failure of BDNF synthesis in the brain^52^. However, data lacks on the expression of BDNF and TrkB in the diabetic GI tract. Lommatzsch et al. has shown that BDNF was highly expressed in the colon, but lacked the expression of both the high- and low-affinity receptors for BDNF, i.e., TrkB and p75^NTR21^. Liu et al. has shown that the levels of some neurotrophic factors such as glia cell-derived neurotrophic factor (GDNF), neurotrophin 3 and nerve growth factor were down-regulated in the colon of diabetic rats^53^. It has been reported that the enteric neuropathy was induced in STZ-induced diabetic rats which is partly mediated via a reduction of GDNF and its main downstream signalling pathway PI3K/Akt^54^. On the other hand, it has been demonstrate that GDNF could increasing the number of NADPH diaphorase–containing neurons in the diabetic intestine through activation of the PI3K/Akt pathway^55^. In the present study, we found that both BDNF and TrkB were highly expressed in the normal colonic wall, whereas such expression was significantly decreased in the diabetic colonic wall. The expressions of BDNF and TrkB were negatively correlated with some morphometric and biomechanical remodeling parameters. The diabetes-induced down-regulation of BDNF and TrkB expression may be associated with diabetes-induced colon dysfunction. BDNF binds to its high-affinity receptor TrkB, resulting in the activation of three different signal transduction cascades^17^: 1) Insulin receptor substrate-1 (IRS-1/2), phosphatidylinositol-3-kinase (PI-3K) and protein kinase B (Akt), 2) Shc/Grb2, Ras, Raf, mitogen-activated protein kinase kinases (MEKs) and extracellular signal regulated kinases (ERKs) and 3) Phospholipase C (PLC), inositol (1,4,5)-trisphosphate [Ins(1, 4, 5)P3], diacylglycerol (DAG) and protein kinase C (PKC). Therefore, the down-regulation of BDNF and its receptor TrkB in the diabetic colon found from the present study may through the same molecular pathway to promote the colon remodeling in diabetes. Future studies are needed to investigate how diabetes induces abnormal expressions of BDNF and TrkB in GI tract. Details on the molecular mechanisms underlying the effect of BDNF and TrkB on diabetes induced GI remodeling are needed to be explored as well.

In the present study we found that the expressions of all proteins - AGE, RAGE, TGF-β1, TGF- β1 receptor, BDNF and TrkB are associated with the glucose level and colon remodeling parameters to some degree. Furthermore, the abnormal expression of these proteins is associated with diabetes-induced complications such as diabetic neuropathy in one way or another. Analysis for interrelations between AGE, RAGE, TGF-β1,TGF- β1 receptor, BDNF and TrkB showed that AGE, TGF- β1 and BDNF in different layers mostly correlated with their receptors RAGE,TGF- β1 receptor and TrkB. This seems to be obvious since the effects of AGE TGF- β1 and BDNF are mediated through their corresponding receptors. However, it is interesting to note that RAGE and TGF- β1 receptor in different layers were strongly correlated each other. There are some studies which have investigated the complicated interaction of AGE and TGF-β1 with their receptors in the pathological progression of diabetic nephropathy^56,57^ and interstitial fibrosis induced by imbalances in extracellular matrix homeostasis^58^. However, to the best of our knowledge, the interplay among these proteins in relation to diabetes-induced GI disorders has not been reported yet and needs to be explored.

In conclusion, STZ-induced diabetes up-regulated the expression of AGE, RAGE, TGF- β1 and TGF- β1 receptor and down-regulated the expression of BDNF and TrkB in different colon layers of rats mainly due to hyperglycemia. The expressions of AGE and TGF- β1 were highly and positively associated with histomorphometric and biomechanical remodeling parameters of colon, and also highly associated with the expressions of their receptors. Therefore, our results suggest that AGE, RAGE, TGF- β1 and TGF- β1 receptor are likely promoting factors for diabetes-induced colon histomorphological and biomechanical remodeling. AGE and TGF- β1 play their roles through their specific receptors RAGE and TGF- β1 receptors. The expression of BDNF and TrkB were highly and negatively associated with histomorphometric and biomechanical remodeling parameters of colon, however we could not conclude that BDNF and TrkB are inhibiting factors or not for diabetes-induced colon remodeling, because novel contribution of BDNF and TrkB in diabetic intestinal dysfunction is scarce. It is needed to further perform mechanistic experiments aimed to study causality or approaches that explain the relevance of the molecular pathways. In the future, it is necessary to investigate the detailed molecular pathway of the abnormal expressions of these proteins in diabetes and their association with diabetes-induced colon remodeling.

## Materials and Methods

### Animal model and groups

Twenty male Sprague-Dawley rats weighing 220-250 g were included in this study. Ten rats were made diabetic by a single tail-vein injection of 40 mg/kg STZ (Sigma-Aldrich, China). After 7 days, this dose of STZ resulted in a random blood glucose level (≥16.7 mmol/L) in 9 of 10 rats which were used for the diabetic group (Diabetes). Another ten rats of similar age and body weight from the same vendor were used as the non-diabetic control group (Control). Approval of the protocol and experimental methods were obtained from The Committee of Guang’anmen Hospital, China Academy of Chinese Medical Sciences for Animal Experimentation.

### Experimental procedures and sampling

The body weight and blood glucose levels were measured at 2-week intervals after the start of the experiment. The experimental period was 60 d. At the end of the experiment, the rats fasted overnight and were then anesthetized with 4% chloral hydrate (10 mL/kg, ip). Following laparotomy, the whole colon was harvested. After the lumen of the segments was gently cleansed with saline, the length and the wet weight were measured. The middle colonic segment was divided into three parts: A 2-cm-long tissue was cut from the proximal end of the segments and fixed in 10% formalin for immunohistochemistry examination. Then a 1-cm-long part was cut and used for the zero-stress state experiment and the remaining part was used for the distension test. The results of zero-stress state and the distension test were reported in our previous paper^22^. Therefore, the parameters of morphometric properties, residual strains and stress-strain of the wall in colonic segments were adopted from our previous paper^22^ and used for correlation analysis for the expressions of different proteins used in the present paper.

### Immunohistochemistry staining

#### Tissue pretreatment

The tissue samples for immunohistochemistry were fixed in 10% phosphate-buffered formalin for 24 h and embedded in paraffin. Five-micron sections were cut perpendicular to the mucosa surface and placed in a water bath at 40 °C. Thereafter, sections were transferred onto pretreated microscopic slides which electrostatically attracted formalin fixed tissue and bond them to the slides. After drying the slides completely at room temperature, they were treated in an oven at 37 °C overnight to enhance the attachment of tissue to the slides. The sections were deparaffinized two times in xylene, 15 min per time, and rehydrated in 100%, 95%, 90%, 80%, 70%, 60% and 50% ethanol two times respectively, 3 sec per time, and were subsequently rinsed 10 min and washed in 0.01 M PBS (pH 7.4).

#### AGE

After treatment with H_2_O_2_ (3% in ethanol, room temperature, 15 min.) and proteinase K (100 µg/ml, 100 µl, 37 °C, 20 min.), the sections were incubated with 5% BSA-PBS buffer (room temperature, 30 min.) in order to block non-specific staining. Afterwards, the sections were incubated with the primary anti-AGE antibody (abcam, 1:100, diluted in 1% BSA-PBS), or normal mouse IgG (250 µg/ml) pre-treated with excessive CML (1:250, diluted in 1% BSA-PBS, negative control) over night at 4 °C. The sections were then washed and incubated with LINK (biotinylated anti-rabbit and anti-mouse immunoglobulin) and STREPTAVIDIN PEROXIDASE (streptavidin conjugated with horseradish peroxidase) respectively at room temperature for 10 min (both are part of reagents of LSAB2 System-HRP, products of Dako Company, Denmark). Then the peroxidase activity was visualized by incubating the sections in substrate working solution containing hydrogen peroxide and 3, 3′-diaminobenzidine tetrahydrochloride at room temperature for 5 min. The sections were rinsed for 10 min, counterstained with Mayer Haematoxylin for 1 min, treated in HCl-ethanol for 3 sec, and dehydrated in 80%, 90%, 95%, 100% ethanol for 3 sec, respectively. Then the slides were immersed in xylene for 15 min two times and mounted.

#### RAGE

The primary anti- RAGE antibody was produced in rabbits immunized with a synthetic peptide corresponding to a sequence at the N-terminal of human RAGE (Sigma). Only two amino acids are different from the related rat sequence. The sections were boiled in 10 mM citrate buffer (pH 6.0) 18 min for retrieving the antigen. Normal rat lungs were used as positive control since RAGE is highly expressed in the lungs^59^. The primary antibody was diluted (1:80) with 1% BSA-PBS and normal rabbit serum (diluted 1:60) pre-treated with excessive soluble RAGE was used as negative control. Other processes were similar to the AGE immunostaining.

#### TGF- β1, TGF- β1 receptor, BDNF and TrkB

The primary antibodies of TGF- β1, TGF- β1 receptor, BDNF and TrkB were obtained from Wuhan Boster Biological Engineering Co., Ltd. They were all diluted (1:100) with 1% BSA-PBS. The second antibody is HRP-Goat Anti-Rabbit IgG and was diluted (1:150) with 1% BSA-PBS. The sections were placed in 3% H_2_O_2_ (AR1108) at room temperature for 5-10 minutes to inactivate endogenous enzymes. The sections were then rinsed with distilled water for 3 times. The sections were immersed in 0.01 M citrate buffer (AR0024, PH6.0) or 0.02 M PBS (AR0030, PH7.2-7.6) and heated to boiling using electricity or microwave in order to retrieve the antigen. This process was repeated one or two times for an interval between 5-10 minutes. Then the slides were naturally cooled to room temperature. The sections were incubated with 5% BSA blocking solution (AR0004) (37 °C, 30 minutes) for blocking non-specific staining. Then the excess liquid was shanked off from the slides (don’t wash) and incubated with diluted primary antibody over night at 4 °C or 2 hours at 37 °C. The slides were rinsed with PBS (pH7.2-7.6) for 3 times (each time lasts 5 minutes). Then the slides were incubated with corresponding second antibodies (37 °C, 30 minutes). The slides were rinsed again for 3 times (each time lasts 5 minutes). The slides were then incubated with SABC (37 °C, 30 minutes) and washed for 4 times (each time lasts 5 minutes) with PBS (pH7.2-7.6). The peroxidase activity was subsequently visualized by incubating the sections with DAB visualized kit (AR1022, taking 1 drop from each of the A, B, C reagents and mixing into 1 ml of distilled water) for about 15 minutes at room temperature. The slides were washed with distilled water and counterstained with hematoxylin (AR0005). At last, the slides were dehydrated, transparent and mounted.

#### Image analysis

AGE, RAGE, TGF- β1, TGF- β1 receptor, BDNF and TrkB are shown to be brown staining, but such color does not appear in the negative control slides, indicating that the staining is specific. To minimize errors, 6 to 10 photographs from different locations of the same layer in each slide were randomly taken, after that, different parts were saved as individual image files. The region of interest (ROI) was defined using Sigmascan Pro 4.0 image analysis software. The color due to 3,3′-diaminobenzidene staining was distinguished in the ROI using intensity thresholds. Finally, the images were exported as binary images. The total area and area fraction of AGE, RAGE, TGF- β1, TGF- β1 receptor, BDNF and TrkB positive staining were calculated by a Matlab program (Matlab 6.5, The MathWorks Inc. USA). Then the fraction of AGE, RAGE, TGF- β1, TGF- β1 receptor, BDNF and TrkB in mucosa, muscle and submucosa layers were computed as: Fraction of protein expressions = immuno-positive area/total measured area.

### Correlation analysis

#### Single linear correlation analysis

As the hyperglycemia is the most common characteristic of diabetic rats, firstly we made linear regression analysis between blood glucose level with all other parameters including morphometry biomechanics and expressions of AGE, RAGE, TGF- β1, TGF- β1 receptor, BDNF and TrkB in different layers of colon wall. In order to analyze the expressions of AGE, RAGE, TGF- β1, TGF- β1 receptor, BDNF and TrkB with the parameters of morphmetry and biomechanics, the single linear regression analysis was done on the expressions of AGE, RAGE, TGF- β1, TGF- β1 receptor, BDNF and TrkB in different layers of the colon wall with body weight, wet weight per unit length of the colon, wet weight of colon to body weight ratio, wall thickness, wall cross-sectional area, opening angle, inner residual strain, outer residual strain, circumferential material constant a and longitudinal material constant a. Furthermore, the single linear regression analysis was also performed in order to examine the correlations between the expression of AGE, TGF-β1 and BDNF in different layers of colon wall with the expressions of their corresponding receptors, i.e., RAGE, TGF-β1 receptor and TrkB respectively.

#### Multiple linear correlation analysis

In order to determine the interrelation among AGE, RAGE, TGF- β, TGF- β receptor, BDNF and TrkB expressions, a multiple linear correlation analysis was done.

### Statistical analysis

The data were representative of a normal distribution and accordingly the results were expressed as means ± SEM. Student’s t-test and analysis of variance (ANOVA) were used to detect differences between parameters and groups (Sigmastat 2.0TM). Linear regression analysis was used to demonstrate eventual association between AGE, RAGE, TGF- β, TGF- β receptor, BDNF and TrkB with histomorphometric and biomechanical parameters. The results were regarded as significant when P < 0.05.

### Data availability statement

HS and JZ had full access to all the data in the study and take responsibility for the integrity of the data and the accuracy of the data analysis.
